# Mortality Rate Among Burn Patients at the Specialized Burns Hospital in Baghdad Medical Complex From 2019 to 2023: A Retrospective Study

**DOI:** 10.7759/cureus.92773

**Published:** 2025-09-20

**Authors:** Batool Qabas, Aya M Khaleel, Ahmed Khalaf Jasim, Luma G Alsaadi

**Affiliations:** 1 Medicine, College of Medicine, University of Baghdad, Baghdad, IRQ; 2 Plastic and Reconstructive Surgery, College of Medicine, University of Baghdad, Baghdad, IRQ; 3 Microbiology, College of Science, Mustansiriyah University, Baghdad, IRQ

**Keywords:** burn injuries, complications of burn injuries, epidemiology of burn injury, iraq, mortality rate

## Abstract

This retrospective study was conducted on patients with burn injuries to assess the extent of damage and evaluate the associated mortality rate in the Specialized Burns Hospital in the Baghdad Medical Complex, Baghdad, Iraq, from 2019 to 2023, and to correlate mortality with age, sex, cause of burn, burn depth, and surface area. A convenient sampling of 593 patients' data was collected using a self-designed form and was taken from the medical records of burn patients at their first discharge. The results showed that the highest mortality rate was among 15-30-year-olds; females had the highest record in comparison to males; 2020 had the highest mortality rate; and second- and third-degree mixed burns had the highest record for mortality rate (50%-75%). The total body surface area (TBSA) burn range of 75%-100% accounted for the highest number of admissions and also had the highest mortality rate. Multiple organ failure was the main complication in comparison to sepsis. Regarding treatment, the mortality rate was 26.7% among patients who underwent surgical intervention and 73% among those admitted to the ICU. All data were collected by the hospital's manual paper-based system instead of a computerized one.

## Introduction

Burns are the most challenging injury that may be encountered. It is associated with severe morbidity and mortality. It’s a common type of skin injury encountered at all levels of medical facilities, from private clinics to core hospitals. Minor burns heal by topical treatment alone, but moderate to severe burns require systemic management and skin grafting [1]. The survival rates for burn patients have made significant strides within the past few decades due to progress in advanced therapeutic care in specialized burn centers [2]. Infection in burn patients is a major cause of morbidity and mortality and remains one of the greatest challenges facing burn teams [3]. Overall, burns result in an estimated 180,000 deaths annually. While rates are decreasing in some high-income countries, burn deaths are significantly higher in low- and middle-income countries, particularly in the African and Southeast Asian regions [4]. This retrospective study aims to clarify the mortality rate among burn patients in the Specialized Burns Hospital at Baghdad Medical Complex, Baghdad, Iraq, from 2019 to 2023, and to correlate mortality with age, sex, cause of the burn, depth of the burn, and surface area.

## Materials and methods

Sample collection

This retrospective study involved a review of patients’ data from their medical charts, including first admission and discharge, at the Specialized Burns Hospital in Baghdad Medical City from 2019 to 2023. Approval was obtained from the hospital administration for data collection, which was conducted in the statistical department using a convenience sample and a self-designed form. The data were collected based on a form that included sex, and year was classified into 2019, 2020, 2021, 2022, and 2023, and age was classified into four categories: 0-15 years, 16-30 years, 31-45 years, and above 45 years.

The type of burn was classified into four categories: thermal (which includes flame and hot liquids), electrical, chemical, and radiation. Degree of burn, which was classified into first, second, third, second and third mixed, and fourth degrees. Total body surface area (TBSA) was classified into four categories: 0%-25%, 25.1%-50%, 50.1%-75%, and 75.1%-100%. Treatment and management were classified into surgical debridement, skin grafts, and intensive care units (ICU). Complications included multiple organ failure, sepsis, and others. Length of hospital stay was classified into three categories: 0 to five days, six to 10 days, and above 10 days. 

Statistical analysis

Patients’ data were collected using a self-designed form that was taken from the medical records of burn patients at their first discharge through five years (from 2019 to 2023). Statistical analysis was performed using IBM SPSS Statistics software, version 29 (IBM Corp., Armonk, NY), using descriptive statistics.

## Results

In this study, the total number of burn patients was 593, with a 32.7% mortality rate. They were divided into four age categories: 0-15 years, which had the highest admission rate at 35.8% (n=212) with a mortality rate of 7.3% (n=43); 16-30 years, which accounted for 33.6% (n=199) and had the highest mortality rate of 14.5% (n=86); 31-45 years, comprising 18.7% (n=111) with a mortality rate of 6.2% (n=37); and above 45 years, which made up 12% (n=71) and had the lowest mortality rate of 4.7% (n=28) (Table 1).

**Table 1:** Mortality rate categorized according to age The highest age-specific mortality rate was observed in the 16–30 age group, accounting for 14.5% of total deaths.

Age group (years)	Total patients	Number of deaths	Overall mortality rate	Mortality rates within categories
0-15	212	43	7.3%	20.2%
16-30	199	86	14.5%	43.2%
31-45	111	37	6.2%	33.3%
>45	71	28	4.7%	39.4%
Total	593	194	32.7%	

The data was collected over a period of five years. In 2019, the total number of patients was 122, with a mortality rate of 7.6% (n=45); in 2020, the number of patients was 121, with the highest mortality rate of 8.4% (n=50); in 2021, the number was 121, with a mortality rate of 6.6% (n=39); in 2022, the number was 112, with a mortality rate of 4.6% (n=27); and in 2023, the number was 117, with a mortality rate of 5.6% (n=33) (Table 2).

**Table 2:** Annual mortality rates during the study period

Year	Total patients	Dead patients	Overall mortality rate	Mortality rate within categories
2019	122	45	7.6%	36.8%
2020	121	50	8.4%	41.3%
2021	121	39	6.6%	32.2%
2022	112	27	4.6%	24.1%
2023	117	33	5.6%	28.2%
Total	593	194	32.7%	

Females had the highest record for admission, which was 337, and the highest mortality rate, which was 23.8% (n=141), and for males, the number was 256 with a mortality rate of 8.9% (n=53) (Table 3).

**Table 3:** Mortality rate categorized according to sex

Sex	Total patients	Dead patients	Overall mortality rate	Mortality rate within categories
Female	337	141	23.8%	41.8%
Male	256	53	8.9%	20.7%
Total	593	194	32.7%	

Regarding the type of burn, cases were categorized into four groups. Thermal burns (including flame and hot liquids) had the highest number of cases (n=548) and the highest mortality rate of 31.9% (n=189); electrical burns accounted for 21 cases with a mortality rate of 0.2% (n=1); chemical burns were reported in nine cases with a mortality rate of 0.7% (n=4); and radiation burns were the least common, with 15 cases and no associated mortality (0%, n=0) (Table 4).

**Table 4:** Mortality rate categorized according to the type of burns

Type of burn	Total patients	Dead patients	Overall mortality rate	Mortality rate within categories
Thermal	548	189	31.9%	34.4%
Electrical	21	1	0.2%	4.7%
Chemical	9	4	0.7%	44.4%
Radiation	15	0	0%	0%
Total	593	194	32.7%	

TBSA was categorized into four groups. A TBSA of 1%-25% had the lowest number of admissions (n=49) and the lowest mortality rate of 2.2% (n=13); 25.1%-50% accounted for 129 admissions with a mortality rate of 4.9% (n=29); 50.1%-75% had the highest number of admissions (n=274) with a mortality rate of 10.1% (n=60); and 75.1%-100% included 141 cases and had the highest mortality rate of 15.5% (n=92) (Table 5).

**Table 5:** Mortality rate categorized according to total body surface area (TBSA)

TBSA	Total patients	Dead patients	Overall mortality rate	Mortality rate within categories
1%-25%	49	13	2.2%	26.5%
25.1%-50%	129	29	4.9%	22.4%
50.1%-75%	274	60	10.1%	21.8%
75.1%-100%	141	92	15.5%	65.2%
Total	593	194	32.7%	

The degree of burns was classified into five categories. First-degree burns accounted for 24 cases with a mortality rate of 0.2% (n=1); second-degree burns included 171 cases with a mortality rate of 1.7% (n=10); third-degree burns had 124 cases with a mortality rate of 11.6% (n=69); fourth-degree burns were reported in 2 cases with a mortality rate of 0.2% (n=1); and mixed second- and third-degree burns had the highest number of cases (n=272) and the highest mortality rate of 19.1% (n=113) (Table 6).

**Table 6:** Mortality rate categorized according to burn degree

Degree	Total patients	Dead patients	Overall mortality rate	Mortality rate within categories
First	24	1	0.20%	4.2%
Second	171	10	1.7%	5.8%
Third	124	69	11.6%	55.6%
Fourth	2	1	0.20%	50%
Second and Third	272	113	19.1%	41.5%
Total	593	194	32.7%	

Regarding complications, these were documented in 274 patients, with an overall mortality rate of 69.5%. Multiple organ failure was the most common complication, occurring in 166 patients, and also had the highest mortality rate of 54.3% (n=149); sepsis was reported in 100 cases with a mortality rate of 14.9% (n=41); and other complications were noted in 8 patients with a mortality rate of 0.3% (n=1) (Table 7).

**Table 7:** Mortality rate classified according to complications

Complication	Total patients	Dead patients	Overall mortality rate	Mortality rate within categories
Multiple organ failure	166	149	54.30%	89.7%
Sepsis	100	41	14.90%	41%
Others	8	1	0.30	12.5%
Total	274	191	69.5%	

Surgical procedures were documented in 173 patients, with an overall mortality rate of 27.16%. These were categorized into two groups: surgical debridement alone, performed in 123 patients with a mortality rate of 24.84% (n=43), and surgical debridement and skin grafting, performed in 50 patients with a mortality rate of 2.31% (n=4) (Table 8).

**Table 8:** Mortality rate classified according to surgical procedures A total of 98 patients required admission to the intensive care unit (ICU), with a mortality rate of 73% (n = 72) among them.

Procedure	Total patients	Dead patients	Overall mortality rate	Mortality rate within categories
Surgical debridement	123	43	24.84%	34.9%
Surgical debridement and skin graft	50	4	2.31%	8%
Total	173	47	27.16%	

Regarding the period of hospitalization, the data were divided into three categories. Hospital stays of 0 to five days had the highest number of cases (n=283) and the highest mortality rate of 15.5% (n=92); six to 10 days included 169 cases with a mortality rate of 11.3% (n=67); and more than 10 days accounted for 141 cases with a mortality rate of 5.9% (n=35) (Table 9).

**Table 9:** The mortality rate classified according to the period of hospitalization

Length of stay	Total patients	Dead patients	Overall mortality rate	Mortality rate within categories
0-5 days	283	92	15.5%	32.5%
6-10 days	169	67	11.3%	39.6%
>10 days	141	35	5.9%	24.8%
Total	593	194	32.7%	

## Discussion

One of the important steps for the evaluation of burn outcomes is the assessment and determination of the mortality rate. There are many predictive parameters that are used to evaluate the mortality rate of burn patients [5].

This study evaluated multiple parameters, including age, sex, cause of burn, duration of hospitalization, and burn-related complications. The overall mortality rate observed was 32.7%. For comparison, Al-Shamsi et al. reported a mortality rate of 22.3% at the Al-Fayhaa Burn Centre in Basra City [6]. Meanwhile, a 2015 study conducted by Lami and Al-Naser at the Specialized Burn Hospital in Baghdad Medical Complex found a mortality rate of 13.3% [7]. In contrast, Bataineh et al. reported a lower mortality rate of 8.1% (43 deaths out of 527 patients) in a study conducted in Jordan [8].

In our study, the highest mortality rate by age was observed in the 16-30-year-old group, at 14.5%, while the lowest mortality rate was seen in patients over 45 years old, at 4.7%. We attribute the elevated mortality in the younger age group possibly to a higher rate of suicide attempts, which is consistent with findings from developing countries. According to Vijayakumar, the suicide rate is notably higher among individuals aged 15-24 years in developed countries, with married females at greater risk [9]. Similarly, Al-Shamsi et al. reported the highest mortality rate in the 15-24-year age group, with 32 deaths out of 93 patients, corresponding to 7.6% [6]. Qader et al. also documented higher mortality rates among patients aged 12-25 years at the Burns and Plastic Surgery Hospital in the Sulaimani region of Kurdistan, Iraq [10]. In addition, Lami and Al Naser found the highest mortality rate of 37.4% in the 21-30-year age group [7]. Conversely, a study conducted in Jordan reported the highest mortality rate (17.6%) among patients older than 40 years [8].

Our finding suggests the mortality rate was higher among females (23.8%), which may be attributed to women spending more time near fire sources and a higher risk of suicide attempts [9]. This finding aligns with the study by Usmani et al., conducted in the Department of Surgery at Mahatma Gandhi Hospital’s Burn Ward, where mortality rates were higher in females (41.94%) compared to males (20.29%) [11]. Similarly, Al-Shamsi et al. reported higher mortality rates in females than males [6]. However, a study by Lami and Al-Naser found higher mortality in males (67%) compared to females (32%), likely due to a significant proportion of male burn patients being personnel injured in battlefield incidents during 2015 [7].

Our results indicate that the leading cause of mortality was thermal burns, including both flame and fluid burns, with a rate of 31.9%. This is consistent with findings from Al-Shammari et al. at King Fahd Hospital and Al-Mutairi et al.'s study at Ministry of National Guard Health Affairs (MNGHA) hospitals in Saudi Arabia, where flame burns were the most common cause of burns [12-15].

Compared to the study by Al-Shamsi et al. in Basra [7], the highest mortality rate was observed in patients with a TBSA of 75%-100%, reaching 87%, while the lowest mortality rate, approximately 1%, occurred in those with a TBSA of 0%-25%. These findings are consistent with our results and align with a study conducted at Mahatma Gandhi Hospital’s Burn Ward in India [11], which reported the highest mortality rate in patients with TBSA over 80% and the lowest in those with TBSA under 20%. In comparison to the findings of Lami et al. [7], which indicated that third-degree burns exhibited the highest mortality rate of 25.7% among 249 patients, followed by mixed injuries (encompassing both second and third degrees) at a rate of 26.1% among 88 patients, and those of Usmani et al. [11], who reported a mortality rate of 47.3% for third-degree (full thickness) burns among 38 patients, followed by 14.52% for second-degree burns among 62 patients, our study presents a contrasting perspective. Our findings suggest that mixed injuries demonstrate the highest mortality rate, followed by third-degree burns, with mortality rates of 19.1% and 11.6%, respectively.

In our study, multiple organ failure was the leading cause of mortality, accounting for 54.3%, followed by sepsis at 14.9%. These results are consistent with those of Lami et al. [7], who reported a 53.3% mortality rate primarily due to multiple organ failure, followed by sepsis at 44.4%. In contrast, Qader et al. [10] found sepsis to be the predominant cause of death, accounting for 55% of mortalities.

In our study, the majority of patients had a hospital stay of 0 to five days, which is comparable to the findings of Sharma et al. in Kuwait, where the median hospitalization duration was six days [13]. In contrast, Bataineh et al. reported a median hospital stay of 16 days [8], and Mahaluxmivala et al. found a median duration of 16.4 days in a study conducted in Saudi Arabia [14]. Notably, in our study, the highest mortality rate was observed among patients hospitalized for five days or less.

Speaking of limitations, one of the causes of death was based on clinical notes written by the treating surgeons, not on official death certificates. This is because death certificates are issued by the coroner’s office in the capital, not by the hospital. Another limitation is that not all patient records were easy to find. This is due to the hospital using a manual paper-based system instead of a computerized one.

## Conclusions

In summary, the majority of burn admissions in Baghdad Medical Complex consist of young adults, children, and females. The primary causes of burns were flame and scald injuries. Death due to burns during the study year was 1:3. Surgical intervention during the study was 1:3. ICU entrance during the study was 1:6. Multiple organ failure is the most prominent cause of death. Half the mortality rate was among patients with TBSA (75-100), which could be attributed to a suicidal attempt, and patients who stayed from 0 to five days. 

## References

[1] Yoshino Y, Ohtsuka M, Kawaguchi M (2016). The wound/burn guidelines - 6: guidelines for the management of burns. J Dermatol.

[2] Church D, Elsayed S, Reid O, Winston B, Lindsay R (2006). Burn wound infections. Clin Microbiol Rev.

[3] El-Kersh DM, Abou El-Ezz RF, Ramadan E, El-Kased RF (2024). In vitro and in vivo burn healing study of standardized propolis: unveiling its antibacterial, antioxidant and anti-inflammatory actions in relation to its phytochemical profiling. PLoS One.

[4] (2023). Burns. https://www.who.int/news-room/fact-sheets/detail/burns.

[5] Pantet O, Faouzi M, Brusselaers N, Vernay A, Berger MM (2016). Comparison of mortality prediction models and validation of SAPS II in critically ill burns patients. Ann Burns Fire Disasters.

[6] Al-Shamsi M, Othman N (2017). The epidemiology of burns in Basra, Iraq. Ann Burns Fire Disasters.

[7] Lami FH, Al Naser RK (2019). Epidemiological characteristics of burn injuries in Iraq: a burn hospital-based study. Burns.

[8] Bataineh Bataineh, Z. A., Al Quran (2018). Pattern of burn injury at north of Jordan. International journal of burns and trauma.

[9] Vijayakumar L (2004). Suicide prevention: the urgent need in developing countries. World Psychiatry.

[10] Qader AR (2012). Burn mortality in Iraq. Burns.

[11] Usmani A, Pipal DK, Bagla H (2022). Prediction of mortality in acute thermal burn patients using the Abbreviated Burn Severity Index score: a single-center experience. Cureus.

[12] Alshammari SM, Almarzouq S, Alghamdi AA, Shash H (2022). Mortality and survival analysis of burn patients admitted in a critical care burn unit. Saudi J Med Med Sci.

[13] Sharma PN, Bang RL, Ghoneim IE, Bang S, Sharma P, Ebrahim MK (2005). Predicting factors influencing the fatal outcome of burns in Kuwait. Burns.

[14] Mahaluxmivala S, Borkar A, Mathur A, Fadaak H (1997). A retrospective study of etiopathological and preventive factors in a burns unit in Saudi Arabia. Burns.

[15] Al-Mutairi AM, Labani S, Alasamari MJ (2023). Burn injury characteristics and outcomes among pediatric and adult patients admitted to Ministry of National Guard Health Affairs (MNGHA) hospitals in Saudi Arabia. Burns Open.

